# A 69-Year-Old Female with Tiredness and a Persistent Tan

**DOI:** 10.1371/journal.pmed.0020229

**Published:** 2005-08-30

**Authors:** Petros Perros

## Abstract

In this case-based learning article, Perros discusses the work- up a woman who presented with palpitations, tiredness, shortness of breath, and a persistent tan.

## DESCRIPTION OF CASE

A 69-year-old female presented with palpitations and a history of tiredness and shortness of breath for several weeks. She had a previous history of Raynaud syndrome. She was an ex-smoker. She commented that she had not lost her tan since the previous summer. Her only medication was nifedipine for her Raynaud syndrome.

On examination, she was slim and tanned. Pulse rate was 86 beats per minute and regular. Her blood pressure (BP) was 105/74 mm Hg. Her chest was hyperinflated. The rest of her examination was recorded as normal. A chest X ray showed no evidence of cardiac failure. Electrocardiogram monitoring showed episodes of atrial fibrillation. Her routine biochemistry was as follows: serum sodium, 132 mmol/l (normal range, 135–145 mmol/l); potassium, 5.1 mmol/l (3.4–5 mmmol/l); urea, 8.6 mmol/l (2.5–6.4 mmol/l); and creatinine, 110 mmol/l (65–120 μmol/l).

She was commenced on digoxin and warfarin. Her breathlessness gradually improved, and she remained in sinus rhythm.

### How Would You Explain the Hyponatraemia, and What Additional Investigations Are Required?

Hyponatraemia is a common electrolyte abnormality in hospitalised patients [1]. The cause is often obvious (e.g., a clear history of fluid and electrolyte loss through vomiting or diarrhoea, or through use of thiazides or loop diuretics). A patient with hyponatraemia should be assessed by first taking a thorough history, focusing on gastrointestinal symptoms, fluid intake, thirst, postural dizziness, and medication. Evidence of intravascular volume depletion should be sought by examining skin turgour, the tongue, jugular venous pressure, and pulse rate, and most importantly, by measuring the BP in the supine and erect positions. A drop of BP by more than 20 mm Hg is indicative of intravascular volume depletion. Measurement of urinary sodium is the single most useful test. A urinary sodium concentration less than 20 mmol/l is indicative of volume depletion due to extrarenal causes (except in certain oedematous states). A urinary sodium concentration greater than 40 mmol/l suggests syndrome of inappropriate ADH secretion (SIADH), or a salt-losing nephropathy (diuretics, primary renal tubular diseases, or adrenal failure). The diagnosis of SIADH requires demonstration of normal thyroid and adrenocortical function, in the absence of intravascular volume depletion [2].

### Progress

**Figure 1:**
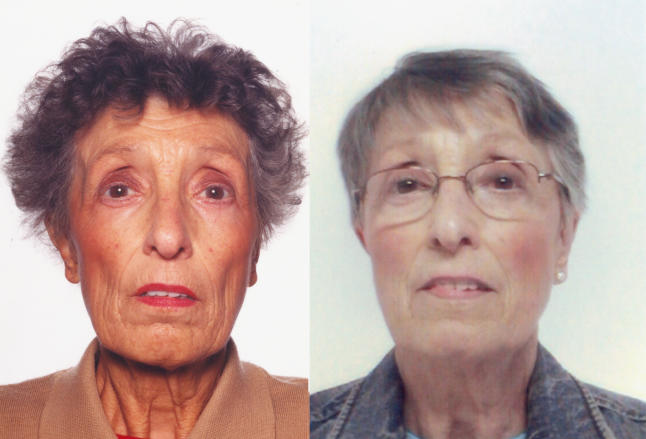
The Patient's Facial Appearance at Presentation versus after Treatment Facial appearance at presentation (left), showing pigmentation, and after treatment (right).

**Figure 2:**
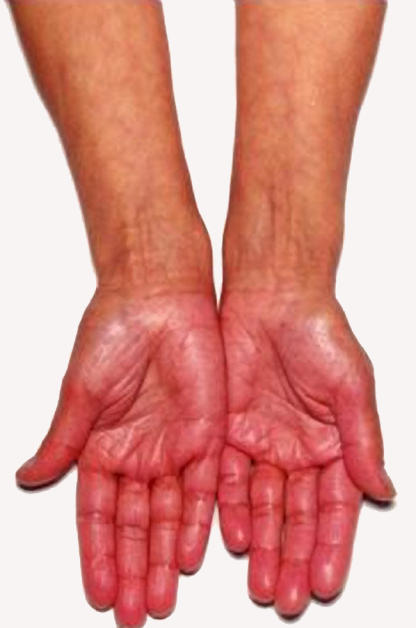
Photo of the Patient's Hand at Presentation, Showing Pigmentation in the Creases

The patient was admitted into hospital two weeks after the initial presentation. Her main complaints were increasing lethargy and tiredness, reduced appetite, an episode of fainting, and weight loss. On examination, she was pigmented and thin (Figures 1 and 2). Her pulse rate was 76 beats per minute in sinus rhythm. BP was 77/59 mm Hg. The rest of the examination was normal.

### What Is the Differential Diagnosis?

A history of malaise, anorexia, and weight loss in a 69-year-old patient is worrisome, and malignancy requires exclusion. She is an ex-smoker; therefore, lung cancer should be considered. SIADH can be a complication of lung cancer, and she has a history of hyponatraemia. Pigmentation may also occur in some lung cancers and other malignancies associated with ectopic adrenocorticotrophic hormone (ACTH) production. However, ectopic ACTH is usually associated with hypokalaemia and hypertension, which have not been features in this patient.

Lung, breast, gastrointestinal, and other malignancies can cause hypercalcaemia due to parathyroid hormone–related peptide secretion, and common symptoms of hypercalcaemia include anorexia, lethargy, and dehydration.

The patient has recently been started on digoxin, and toxicity from this drug can manifest as anorexia and malaise. The recent introduction of warfarin may also have led to occult blood loss, which could account for her malaise and hypotension.

Generalised pigmentation can be a manifestation of other systemic disorders. The patient is known to have Raynaud syndrome, and systemic sclerosis is associated with both Raynaud and pigmentation but is rare, and she has no other features, such as arthropathy or vasculitic lesions. Haemochromatosis can cause pigmentation and present with malaise due to liver failure, diabetes, or other rarer endocrinopathies, and predisposes a patient to hepatocellular carcinoma. Thyrotoxicosis and type 1 diabetes sometimes present in an atypical manner in patients of this age group, and need to be excluded. Primary adrenal failure could explain the patient's anorexia, malaise, pigmentation, low blood pressure, fainting, and serum electrolytes.

If you suspected primary adrenal failure, the specific symptoms that you would ask about are shown in Box 1.

**Box 1. Common Symptoms of Primary Adrenal Failure**

- Tiredness
- Anorexia
- Nausea and vomiting
- Abdominal pain
- Weight loss
- Postural dizziness
- Pigmentation
- Craving for salt
- Weakness
- Hypoglycaemic episodes

### Investigations following the Second Hospital Admission

Following the second hospital admission, the chest X ray was unchanged from previous, with no masses. Other investigations had the following results: electrocardiogram, sinus rhythm, no digoxin effect; creatinine, 87 mmol/l (65–120 μmol/l); plasma glucose, 3.4 mmol/l; potassium, 5.4 mmol/l (3.4–5 mmol/l); sodium, 113 mmol/l (135–145 mmol/l); urea, 5.2 mmol/l (2.5–6.4 mmol/l); urine sodium, 45 mmol/l; serum thyroid-stimulating hormone, 7.87 mU/l (0.3–4.1 mU/l); serum free thyroxine, 18 pmol/l (11–23 pmol/l). Serum calcium, haemoglobin, and liver function tests were normal. Ultrasound scan of the abdomen was normal. Autoantibodies and extractable nuclear antigen Scl70 were negative; extractable-nuclear-antigen ribonucleic proteins were positive; thyroid microsomal antibodies were positive with a titre greater than 1/800; and antinuclear antibodies were negative.

### How Would You Manage the Patient's Hyponatraemia?

This patient now has developed severe hyponatraemia and is at risk of developing neurological problems (deterioration in level of consciousness, and fits); therefore, treatment is required as a matter of urgency. Correct management depends on the cause. If the diagnosis is SIADH, the patient needs to be fluid restricted; if it is volume depletion, she will require intravenous fluid and electrolytes.

The low BP and high potassium are against a diagnosis of SIADH. It can be argued that salt and water depletion that is sufficient to cause severe hyponatraemia would be expected to be associated with a higher level of urea. Serum urea, however, is also dependent on the patient's muscle mass, which in this case was low. SIADH usually is associated with a urea level of less than 4 mmol/l. The high level of urine sodium is consistent with SIADH, but also with salt loss from the kidneys.

The diagnosis, therefore, is volume depletion due to renal losses, and normal saline should be administered intravenously.

The rapidity by which hyponatraemia is corrected is crucial. If the serum sodium is corrected too quickly and the hyponatraemia is chronic, there is a risk of central pontine myelinolysis [3]. Frequent monitoring of serum sodium, aiming for a rise of serum sodium of no more than 10 mmol/l/d, should be undertaken when the duration of hyponatraemia is chronic or unknown [2]. In patients where the hyponatraemia is known to have developed within the previous 2–3 days, it can be corrected fast, if there is an indication for doing so (coma or fits).

### How Would You Explain the Other Laboratory Abnormalities?

Antibodies to extractable-nuclear-antigen ribonucleic protein are associated with mixed connective tissue disorder, but the specificity of the test is only 60%–75% [4]. The diagnosis of mixed connective tissue disorder requires additional clinical features that this patient did not have, although the positive antibodies to extractable-nuclear-antigen ribonucleic protein indicate that this patient has an autoimmune predisposition.

The patient's thyroid blood tests show a raised serum thyroid-stimulating hormone and normal free thyroxine, a condition often referred to as “subclinical” hypothyroidism. Her thyroid microsomal antibodies are also markedly positive and suggest an underlying autoimmune thyroiditis.

### What Tests Are Required to Confirm Primary Adrenal Failure (Addison Disease)?

The biochemical diagnosis of Addison disease is made by measuring the serum cortisol concentration. Cortisol is a stress hormone with a prominent diurnal variation, consisting of an early morning peak and low levels in the evening and early part of the night. A single, random cortisol measurement, therefore, may not be diagnostic, unless the patient is physiologically stressed at the time (for example, patients presenting with an adrenal crisis can be diagnosed by a random cortisol measurement taken at the time of their illness and before steroid therapy is initiated). Hypotensive patients with an intact adrenal axis are expected to have a serum cortisol greater than 550 mmol/l; therefore, a cortisol concentration significantly lower than that, in such circumstances, is inappropriate. A blood sample should also be taken for plasma ACTH, which will indicate whether the hypoadrenalism is due to primary adrenal disease (high ACTH) or hypothalamic/pituitary disease (low ACTH). Measurement of the plasma renin activity and aldosterone concentrations is also helpful: in Addison disease plasma renin activity is high (because of the low intravascular volume) and plasma aldosterone is low (because of inability of the adrenal cortex to produce it).

In more stable or ambulant patients, a short synacthen test is usually diagnostic. The criterion for a normal synacthen test is a 30-minute cortisol value of more than 550 mmol/l [5]. The protocol for a short synacthen test is shown in Box 2.

**Box 2. Protocol for Short Synacthen Test**

1. Test is best performed in the morning, e.g., at 9:00 am.
2. Insert intravenous cannula.
3. Take basal cortisol, ACTH, renin, aldosterone, urea and electrolytes, and adrenal antibodies.
4. Short synacthen, 250 μg, is given as intravenous bolus at time zero.
5. Take samples at 0, 30, and 60 minutes for cortisol.

This patient had a short synacthen test, and the results were as follows: cortisol at baseline, 135 mmol/l; cortisol at 30 minutes, 144 mmol/l; baseline ACTH, 434 pg/l (0–47 pg/l); plasma renin activity, 8.9 ng/ml/h (1.1–4.1 ng/ml/h); and aldosterone, <50 pmol/l (220–430 pmol/l).

### What Steroid Regimen Should the Patient Have at This Stage?

Patients in adrenal crisis or those who are hypotensive or cannot take medication orally should have parenteral glucocorticoid, in addition to saline and dextrose. In such a scenario, hydrocortisone is administered intravenously or intramuscularly by multiple bolus injections or by continuous infusion. A common regimen is 100–150 mg of hydrocortisone daily, until the patient's condition improves [6]. With such supraphysiological doses of glucocorticoids, no mineralocorticoid is required. If the patient is not acutely ill and is able to take steroids orally, hydrocortisone may be given initially at a dose of 40 mg in the morning and 20 mg in the evening. Once the patient's condition is improved (usually 3-4 days), the dose of hydrocortisone is reduced to 20–30 mg daily in two or three divided doses, with one-half to two-thirds of the dose taken on rising in the morning and the rest at noon and in the early evening. Mineralocorticoid is usually also introduced at this stage (fluodrocortisone 50–100 mg daily, as a single dose).

### What Additional Investigations Are Required?

The cause of Addison disease needs to be identified. Autoimmune adrenalitis is the most common cause in the developed world, but infectious diseases like tuberculosis and fungal infection are common in some areas [7]. In an older age group, metastases to the adrenal glands are a possibility. Haemorrhage into the adrenals or infarction can occur in the context of meningococcal septicaemia, overanticoagulation, and antiphospholid syndrome.

In this case, adrenal antibodies were strongly positive and an ultrasound of the abdomen showed no abnormalities; therefore, more investigations were not undertaken. If imaging of the adrenals is specifically indicated, computerised tomography scanning is the best modality.

### Should the Hypothyroidism Be Corrected by Adding Thyroxine?

Autoimmune hypoadrenalism and thyroid disease often coexist. It is imperative that the adrenal failure is treated first, as thyroxine replacement in an undiagnosed patient with Addison disease can precipitate a crisis [8]. Often, mild abnormalities in thyroid function (subclinical hypothyroidism) resolve after initiation of steroid therapy without thyroid replacement, as was the case in this patient.

## DISCUSSION

Addison disease usually presents with nonspecific symptoms and should be considered in patients with unexplained malaise. Pigmentation, when present, is an important clue to the diagnosis. Hyponatraemia is almost invariable, though it may be mild and easily missed, as it is a common finding in hospitalised patients. Hyponatraemia merits investigation to reveal the underlying cause, as the treatments may be very different—fluid restriction if due to SIADH and fluid administration if due to fluid and electrolyte depletion. If the hyponatraemia is chronic, correction should be slow and closely supervised. Subclinical hypothyroidism may coexist with Addison disease. As thyroid disease is much more common than Addison disease and thyroid function tests are frequently requested, the patient's symptoms may be attributed to the thyroid dysfunction. Initiation of thyroxine therapy in such cases can precipitate an adrenal crisis, and physicians should think about Addison disease in patients whose symptoms deteriorate after thyroxine.

**Key Learning Points**

- Think of Addison disease in patients complaining of tiredness and who look genuinely ill.
- Check supine and erect BP in hyponatraemic cases.
- The most useful biochemical test in assessing hyponatraemia is the urine sodium.
- The diagnosis of SIADH requires exclusion of adrenal and thyroid failure.
- An inappropriately low serum cortisol in a critically ill patient may be diagnostic of hypoadrenalism.
- The definitive diagnostic test for Addison disease is the short synacthen test.

Once primary adrenal failure is diagnosed, look for the underlying cause.

## Abbreviations

ACTH: adrenocorticotrophic hormone
BP: blood pressure
SIADH: syndrome of inappropriate ADH secretion
